# Cooking and Meal Planning as Predictors of Fruit and Vegetable Intake and BMI in First-Year College Students

**DOI:** 10.3390/ijerph16142462

**Published:** 2019-07-11

**Authors:** Andrea J. Hanson, Kendra K. Kattelmann, Lacey A. McCormack, Wenjun Zhou, Onikia N. Brown, Tanya M. Horacek, Karla P. Shelnutt, Tandalayo Kidd, Audrey Opoku-Acheampong, Lisa D. Franzen-Castle, Melissa D. Olfert, Sarah E. Colby

**Affiliations:** 1 Department of Health and Nutritional Sciences, South Dakota State University, Box 2203, Wagner 425, Brookings, SD 57007, USA; 2 Business Analytics and Statistics, University of Tennessee, 916 Volunteer Boulevard, SMC 247, Knoxville, TN 37996-0532, USA; 3 Department of Nutrition, Dietetics, and Hospitality Management, Auburn University, Poultry Science Building 102A, Auburn, AL 36849, USA; 4 Department of Public Health, Food Studies and Nutrition, Syracuse University, 588 White Hall, Syracuse, NY 13244-3240, USA; 5 Family, Youth and Community Sciences, University of Florida, Box 110310, McCarty Hall 3038A, Gainesville, FL 32611-0310, USA; 6 Department of Food, Nutrition, Dietetics and Health, Kansas State University, 1324 Lovers Lane, Justin Hall 203, Manhattan, KS 66506, USA; 7 Nutrition and Health Sciences Department, University of Nebraska-Lincoln, Ruth Leverton Hall 110, Lincoln, NE 68583-0806, USA; 8 Human Nutrition and Foods, Division of Animal and Nutritional Sciences, School of Agriculture, West Virginia University, 333 Agriculture Science Building, G028, Morgantown, WV 26506, USA; 9 Department of Nutrition, University of Tennessee, 1215 W. Cumberland Ave, 229 Jessie Harris Building, Knoxville, TN 37996, USA

**Keywords:** fruit and vegetable intake, Body Mass Index, cooking, dietary behaviors, meal-planning behaviors

## Abstract

The objective was to determine if cooking skills and meal planning behaviors are associated with greater fruit and vegetable intake and lower body mass index (BMI) in first-year college students who are at risk for excessive weight gain. A cross-sectional analysis was conducted using baseline data from a multi-state research project aimed at preventing weight gain in first-year college students. Cooking type, frequency and confidence, self-instruction for healthful mealtime behavior intention, self-regulation of healthful mealtime behavior, and cup equivalents of fruits and vegetables (FV) were measured using validated surveys. BMI was calculated from measured height and weight. First-year students (*n* = 1108) considered at risk for weight gain from eight universities completed baseline assessments within the first month of entering college. Multiple linear regression was used to determine associations among independent variables of cooking patterns, meal planning behaviors, and dependent variables of fruit and vegetable intake and BMI, after controlling for the influence of sex. Cooking more frequently, cooking with greater skills, and practicing meal planning behaviors are associated with greater fruit and vegetable intake and lower BMI in first-year college students. Interventions aimed at improving health in college students may be enhanced by incorporating cooking and meal planning components.

## 1. Introduction

Greater than one-third of the adults in the U.S have a body mass index equal to or greater than 30, classifying them as obese [1]. Obesity is associated with chronic diseases as diabetes, heart disease, and some cancers [2,3]. Obesity in young adults has tracked into adulthood [1]. Gordon-Larsen et al. [4], have reported that a high proportion of obese adults were previously obese as adolescents (ages 13–20), and only a small proportion of adolescents moved out of the obese category (to overweight or normal weight) as they became adults [4]. Unhealthy behaviors like poor dietary intake, decreased physical activity, inconsistent sleep patterns, and increased sedentary time that have been associated with the development of obesity are often learned at a young age and tend to continue through adolescence into adulthood [5]. Promotion of healthful behaviors that support the prevention of excessive weight gain in young adults is important for obesity prevention and, thus, the chronic diseases associated with obesity. 

Young adults are also at greater risk for experiencing unintended weight gain as the transition between high school and college presents many opportunities for lifestyle changes [6,7]. On average, young adults attending college gain 0.7 kg (1.6 lbs.) to 4.0 kg (8.8 lbs.) during their first year [8]. Although this initial weight gain may occur, weight gain often persists beyond the first year and continues throughout the duration of one’s college experience [9,10]. This trend of weight gain may negatively impact health and weight outcomes in later adulthood. The first year of college may represent a ‘tipping point’ in lifestyle behavioral patterns that persist into adulthood [9]. 

Furthermore, this weight gain may be even more detrimental to those students from families of lower economic status, such as those from ethnic minorities and first-generation college status. First-generation college and ethnic minority students are more likely to come from families of lower economic status with limited access to information about college experiences; thus, transition to college life may be more difficult [11]. It has been reported that socioeconomic status is inversely correlated with body mass index [12]. 

Young adults attending college experience major lifestyle changes often relating to poor dietary intake. Increased consumption of fatty foods and decreased consumption of fruits and vegetables, whole grains and lean proteins are directly related to weight gain and adiposity [13]. The American College Health Association National College Health Health Assessment of Fall 2018 [14] reported that 95.8% of college students surveyed did not consume the recommended five servings of fruits and vegetables daily. Lack of adequate fruit and vegetable intake has been reported to be associated with poor diet quality and excessive weight gain [13]. It has also been reported that cooking skills influence diet quality [15].

Reported barriers to healthy eating include availability and accessibility of food and the resources necessary to prepare food, lack of knowledge or skills in cooking and nutrition, lack of time, and lack of motivation to live healthily [16,17]. It has been reported that those with greater food preparation and cooking skills have diets of better quality [15,16,18]. However, there is limited evidence on the influence of food preparation skills and fruit and vegetable intake in young adults. Therefore, the purpose of this study is to determine if cooking skills and meal planning behaviors are associated with increased fruit and vegetable intake and lower body mass index (BMI) in college students who are at risk for excessive weight gain. It is hypothesized that students’ cooking patterns and meal planning behaviors will be associated with increased fruit and vegetable intake and more healthful BMI. 

## 2. Methods

### 2.1. Study Design

Data were collected through the GetFRUVED research project at baseline on participants in the fall of 2015. GetFRUVED is a community-based participatory research project utilizing social marketing and environmental change to promote health and prevent unintended weight gain among older adolescents entering college who are at risk for excessive weight gain [19]. Eight universities collected data at baseline at their respective university, and freshman students that were assessed from all universities were included in the dataset for this cross-sectional study. Physical measurement assessments were collected by trained researchers at each campus and questionnaires were administered via computers. 

### 2.2. Participants

Potential participants were recruited through new student and freshman orientation, fliers, tabling, promotional item giveaways, e-mails, class presentations, and postcards from approximately 32,000 potential students at the eight universities. Interested participants were required to complete a short eligibility survey. Eligibility criteria required all participants to be first-year college students at least 18 years of age or older attending a participating GetFRUVED university and consuming less than two servings of fruits or three servings of vegetables daily, on average. Students were also required to meet at least one additional eligibility criterion: (1) having a BMI ≥ 25 kg/m^2^ based on participant’s self-reported height and weight; (2) self-identifying as a first-generation college student; (3) identifying his/her parent(s) as overweight or obese; (4) coming from a low-income background measured through an affluence scale; or (5) self-identifying as a racial minority. Individuals meeting eligibility criteria were invited to participate in the study. Of the 5413 incoming freshman who took the screener, 2750 were determined to be at risk and eligible for a full assessment. Only freshman participants (*n* = 1108) completing all assessments (survey and anthropometrics) were included in the dataset for analysis. Data were collected from participants at baseline within the first month of being on campus. For this cross-sectional study, Institutional Review Board (IRB) approval was obtained at all universities in accordance with the policy statements of the Human Subjects Committee. All participants provided written informed consent.

### 2.3. Assessments

#### 2.3.1. Fruit and Vegetable Intake

Fruit and vegetable intake was measured using the National Cancer Institute’s Fruit and Vegetable Screener [20]. Questions queried how much and how often participants consumed various fruits and vegetables over the last month. A final score was calculated as cup equivalents of fruits and vegetables (FV) and presented as mean ± SD [21]. Five participants reporting non-plausible intakes greater than or equal to 15 cups of fruit and vegetables per day were excluded from data analysis.

#### 2.3.2. Body Mass Index

BMI was calculated from measured height and weight using the standard metric equation. Height and weight were assessed by trained researchers. Each measurement was taken twice and the average was recorded. Height was recorded to the nearest 0.1 cm using a SECA 213 portable stadiometer (Seca North America, Chino, CA, USA). Both measurements were required to be within 0.2 cm of each other, or a third measurement was taken. Weight was measured using a SECA digital scale and recorded to the nearest 0.1 kg. Measurements were within 0.2 kg of each other or a third measurement was collected. All instruments were calibrated prior to all assessments. 

#### 2.3.3. Cooking Frequency, Type, and Confidence

Cooking frequency, type, and confidence were measured using a short survey originally developed to assess the impact of cooking interventions [22]. Cooking frequency was assessed using one question asking how often per week participants prepared meals from basic ingredients such as combining ground beef, tomato sauce, cheese, and noodles to make lasagna. Response choices were (1) daily, (2) 4–6 times weekly, (3) 2–3 times weekly, (4) once weekly, (5) less than once weekly, and (6) never [19]. For data analysis, cooking frequency was divided into three categories—‘0× weekly’, ‘1–3× weekly’, and ‘4–7× weekly’, representing how often participants prepare and cook a main meal from basic ingredients. 

To assess cooking type, participants were asked to mark yes or no to completing any of the following types of cooking activities: (1) cooking convenience foods and ready-made meals; (2) combining ready-made ingredients to make a complete meal; (3) prepare dishes from basic ingredients; (4) other; or (5) do not cook at all. For the ‘other’ responses, participants were asked to describe the ‘other’ methods of cooking they perform. ‘Other’ (written) methods were converted to the existing response options to be included in existing categories. Written responses such as ‘dining hall’ or ‘family cooks’ were converted into ‘do not cook at all’ while ‘microwavable meals’ or ‘frozen meals’ became cooking convenience foods and ready-made meals. The cooking type responses (‘do not cook at all’, ‘cooking mostly convenience foods and ready-made meals’, and ‘prepare dishes from basic ingredients’) were assigned numerical scores from least (1) to greatest (3) level of complexity in cooking with ‘do not cook at all’ as least and and ‘prepare dishes from basic ingredients’ as greatest. An individual marking multiple method of cooking was categorized based on the most complex level of cooking they reported. For example, an individual reporting they cook by combining ready-made ingredients to make a complete meal and prepare dishes from basic ingredients would be categorized in the category ‘preparing dishes from basic ingredients’. 

Cooking confidence was measured using a seven-point Likert scale with four different questions addressing how confident participants felt about practicing various cooking techniques such as being able to cook from basic ingredients, following a recipe, tasting food they have not eaten before, and preparing and cooking new foods and recipes [22]. Responses were (1) extremely confident; (2) very confident; (3) moderately confident; (4) neutral; (5) slightly confident; (6) not very confident; and (7) not at all confident [22]. ‘Choose not to answer’ was also an option. Each confidence variable was reverse coded to produce a scale with higher scores indicative of greater confidence (1 = not at all confident, 7 = extremely confident). The average score of all four questions was calculated for an overall cooking confidence score. Cronbach’s alpha was calculated to ensure internal consistency of the calculated variable [23,24]. Cronbach’s alpha was calculated to be 0.89, signifying good internal reliability. 

#### 2.3.4. Self-Instruction for Intention of Healthful Mealtime Behavior and Self-Regulation for Healthful Mealtime Behavior

Self-instruction for intention of healthful mealtime behavior and self-regulation of healthful mealtime behaviors were assessed using survey questions developed for Project YEAH [7,25,26]. to capture how often in the past three months they had intended and/or practiced behaviors like planning, choosing and assembling healthful meals. Self-instruction for intention of healthful mealtime behavior was measured using six questions with responses scaled from never (1) to always (5) on a five-point Likert scale. The participant was asked to indicate how often in the past three months they had: (1) reminded myself that planning quick and simple meals is important; (2) told myself that healthy meals do not require a lot of work; (3) reminded myself to eat in moderation; (4) told myself to allow room for an occasional treat food or dessert for just plain enjoyment; (5) reminded myself to think about my beverage choices; and (6) told myself that fruits and vegetables should be included in every meal. The average score of all six questions was calculated for an overall self-instruction for health mealtime score. Similarly, self-regulation for healthful mealtime behavior (MB) were measured with four questions asking participants to indicate how often in the past three months they had practiced the noted mealtime behaviors including: (1) planned quick, easy, and healthy snacks; (2) selected beverages with health in mind; (3) purposely added vegetables to meals and snacks; and (4) been flexible and sensible in food choices. The responses were scaled from never (1) to always (5) on a five-point Likert scale. The average score of all four questions was calculated for an overall mealtime behavior score. Cronbach’s α for self-instruction for healthful mealtime behavior and self-regulation for healthful mealtime behavior were 71 and 73, respectively. 

### 2.4. Data Analysis

Stata 13.1 (2013) statistical software (StataCorp LP., College Station, TX, USA) was used for all data analyses. The Shapiro–Wilk W test was used to examine the normality of dependent variables fruit and vegetable intake and BMI. The hypothesis of normality was rejected for both variables (*p* < 0.001), and a log transformation was applied to both. Multiple linear regression was used to determine associations among independent variables of cooking patterns, meal planning behaviors and log-transformed dependent variables of fruit and vegetable intake and BMI, after controlling for the influence of sex. While multiple variables including age, ethnicity, residency location, university, working hours, and sex were examined for their relationship with the outcome variables of fruit and vegetable intake and BMI, only sex was significant, hence its inclusion in the final regression analyses. Separate models were run for each log-transformed dependent variable (fruit and vegetable intake and BMI) and independent variable of interest (cooking patterns and meal planning behaviors) and statistical significance was set at *p* ≤ 0.05.

## 3. Results

Surveys and anthropometric measurements were collected from 1108 participants at baseline of the GetFruved project [19]. A comprehensive demographic description of the participant population is presented in Table 1. Participants were 18.5 ± 0.6 years old with 53.9% White, 10.5% Black/African American, 3.1% Hispanic/Latino, and 32.6% other (including biracial). Mean fruit and vegetable cup equivalents was 2.4 ± 2.0 cups/day and BMI was 24.4 ± 4.9. 

### 3.1. Fruit and Vegetable Intake

Cooking frequency of 4–7 times per week was associated with fruit and vegetable intake (β = 0.26, *p* = 0.004). There was no association between less frequent cooking (1–3 times weekly) and fruit and vegetable intake. Within cooking type, cooking mostly convenience and ready-made meals was negatively associated with fruit and vegetable intake (β = −0.14, *p* = 0.009). Cooking from basic ingredients was not associated with fruit and vegetable intake (*p* = 0.392). Cooking confidence was not associated with fruit and vegetable intake (*p* = 0.055). Both self-instruction for intention of healthful mealtime behavior (β = 0.24, *p* = 0.000) and self-regulation for healthful mealtime behavior (β = 0.34, *p* = 0.000) were associated with fruit and vegetable intake (Table 2).

### 3.2. Body Mass Index

Cooking from basic ingredients (β = −0.03, *p* = 0.044) and self-regulation for healthful mealtime behavior (β = −0.01, *p* = 0.033) were significantly associated with BMI (Table 2). There were no associations with cooking frequency, cooking mostly convenience and ready-made meals, cooking confidence, or self-instruction for intention for healthful mealtime behavior with BMI (Table 2). 

## 4. Discussion

Preventing excessive weight gain by implementing healthful dietary behaviors is important for preventing obesity and lifestyle induced chronic diseases. It has been reported that those with greater food preparation and cooking skills have diets of better quality [15,16,18], supporting the need for interventions preventing excessive weight gain, especially in young adults. Since foods are often not consumed exclusively for the nutrient composition, there are many factors that can be associated with an individuals’ dietary intake [27]. This study identified behaviors that were likely associated with fruit and vegetable intake and BMI in first-year college students. In this cross-sectional analysis completed on nutritionally-vulnerable college students, type of cooking and cooking frequency, self-instruction for intention of healthful mealtime behaviors and self-regulation for healthful mealtime behaviors were predictors of fruit and vegetable intake. Cooking from basic ingredients and self-regulation for healthful mealtime behaviors were predictors of BMI. The BMI was lower in the participants who indicated greater frequency of these two behaviors. 

In the present study, cooking 4–7 times per week and more frequently preparing meals from basic ingredients was associated with greater fruit and vegetable intake. Cooking using mostly convenience foods and ready-made meals was negatively associated with fruit and vegetable intake, while cooking from basic ingredients was negatively associated with BMI. Ready-made and convenience foods, are often rich in energy, fat, salt, and sugar but lack the recommended servings of fruits and vegetables [16]. Others have reported similar findings. A cross-sectional study conducted by van der Horst et al. concluded that overweight participants were more likely to consume ready meals compared with normal weight participants [16]. Other factors that were positively associated with BMI were perceiving ready-made meals as healthy and enjoying the taste of convenience foods [16]. Larson et al. reported that young adults more frequently participating in food preparation and less frequently consuming fast food were more likely to meet the dietary objectives of Healthy People 2010 for fat, calcium, and fruit and vegetables [15]. 

The current study also explored cooking confidence as a possible factor influencing dietary intake and BMI; however, there was not significance relationship of cooking confidence with neither dietary intake of fruit and vegetables nor BMI. There is limited research currently addressing cooking confidence, but a critical review assessing the theory of planned behavior notes that self-efficacy is often viewed as a concept related to behavior [28]. Theoretically, the more individuals believe in their capability to achieve different levels of performance, the more likely behavior change will happen [29]. The more confidence an individual has in his/her ability to cook, read a recipe, or try new foods, the more likely he/she is to frequently participate in these activities. This lack of concordance between cooking confidence and dietary intake merits further exploration. 

Self-instruction for intention of healthful mealtime behavior (i.e., planning for healthful meals and snacks) and self-regulation for healthful mealtime behavior (i.e., self-reported healthful meal-time behavior) predicted greater fruit and vegetable intake in this sample of young adult college students. Additionally, those with higher scores for self-regulation for healthful mealtime behavior had lower BMIs. Few studies examine the specific relationship of these variables to dietary intake and health, but Ducrot et al. [30] notes that compared to individuals that do not practice meal planning behaviors, meal planners were more likely to better adhere to the dietary guidelines and consume a variety of food. Meal planning was also associated with less likelihood of overweight an obesity in women, and obesity in men [30]. Meal planning behaviors examined in the study included frequency of planning meals ahead of time, grocery shopping and cooking [30]. Ducrot et al. also concluded that only small differences existed in overall energy and nutrient intake between individuals who practice meal planning versus those who do not [30]. However, fruit and vegetable intake was greater in those that frequently practiced meal planning behaviors [30]. Another study by Laska et al. [31] found that emerging adults (19–23 years) that practiced food preparation, were more likely to have better diet quality five years in the future including increased fruit and vegetable intake and decreased consumption of sugar-sweetened beverages. Research also indicates that diet quality may be related to time spent preparing food [31]. Spending more time on food preparation at home is associated with indicators of higher diet quality, including increased vegetable and fruit intake [32]. The theory of planned behavior also suggests that intention is an important attitude toward action [29]. An individual’s intention to perform (or not perform) a specific action or behavior is the immediate, most significant determinant of that action [28]. While the theory of planned behavior provides theoretical evidence to support the relationship between dietary intake, BMI, and meal planning behaviors, more research is needed [28,29]. 

Previous research also suggests that healthy behaviors tend to cluster together. In a study by Colby et al. [33], individuals that reported more healthful behaviors consumed more fruits and vegetables, had greater intention for meal planning and mealtime behaviors, consumed less fat and calories from sugar-sweetened beverages, reported greater physical activity, and had a lower average BMI compared to more at-risk individuals [33]. Another analysis using Project WebHealth data reported similar findings, suggesting that individuals reporting more healthful behaviors had greater fruit and vegetable intake and physical activity, and lower BMI than at-risk individuals of the same sex [34]. Although the current study was not a cluster analysis and the level of physical activity, amount of fat and calories from sugar sweetened beverages is not reported in this manuscript, certain healthful behaviors did show association with fruit and vegetable intake and BMI. Individuals who indicated use of more complex cooking skills and meal planning behaviors were related to greater fruit and vegetable intake and lower BMI. 

With the positive association of meal planning behaviors and cooking more frequently, including classes on meal preparation skills in wellness interventions maybe beneficial in improving diet quality and healthier food choices. Utter et al. reported in a longitudinal study of teens and young adults that those who reported greater perceived adequacy of cooking skills at 18–23 years of age had greater odds of having healthful dietary behaviors at ages 30–35 years of age [35]. However, the mode of the delivery of the lessons and frequency may determine if a behavior change occurs. Clifford et al. reported that four weekly episodes of an on-line cooking show increased knowledge about healthful dietary behaviors, but did not change behavior in off-campus college students [36]. Levy and Auld reported that four 2 h cooking classes and a supermarket tour to college students enhance the attitude towards cooking and increased cooking confidence, but did not change dietary behavior [37]. In contrast, Bernando et al. demonstrated that a six-week healthy cooking intervention (weekly meetings of 3 h each) increases in cooking confidence for basic cooking techniques and use of fruits and vegetables, accessibility and availability of fruits and vegetables in the home, self-reported knowledge of cooking terms and techniques, and decreases in consumption of fast-food [38]. 

Strengths of this study were its large, diverse sample and use of a comprehensive evaluation of fruit and vegetable intake using validated evaluation tools (NCI Fruit and Vegetable Screener). This study also used measured height and weight, eliminating risk of false self-reporting. However, study limitations should also be considered when interpreting results. Inclusion criteria for GetFRVUED were specific and only those at risk for unhealthful behavior were included. Measuring behaviors by self-report may have also been influenced by social desirability. Although a validated questionnaire was used, the questions assessing cooking patterns could be a limitation due to the timing of survey administration. The survey was administered upon arrival to campus and queried ‘how often in the past month’ participants engaged in specific tasks and consumed certain products. Responses relied on the students’ understanding of the that the timeframe being assessed was the time prior to coming to campus. Additionally, this was a study of association only, and that the causal pathway remains conjectural at this point. Students more attentive to their weight and health overall, for instance, may also be more motivated to learn cooking skills and prepare their own meals. Although this was a geographically diverse sample, the recruitment was through convenience sampling, thus, results may not be generalizable to all college students. 

## 5. Conclusion 

Behaviors adapted and learned during adolescence and as a young adult are likely to persist into adulthood and poor health behaviors can result in negative health outcomes. Identifying lifestyle behaviors associated with dietary intake and BMI in young adults at-risk for weight gain is important because interventions to improve lifestyle could result in health benefits that improve quality of life and reduce disease risk. Interventions aimed at improving dietary intake and BMI could benefit from including food preparation instruction especially those that include cooking and meal planning skills and behaviors. 

## Figures and Tables

**Table 1:** Demographics of first-year college students.

Variable	*n* (%) or Mean ± SD (Range)
Age, *n* (%)	
18–19	1095 (98.8%)
20 and older	13 (1.2%)
Ethnicity, *n* (%)	
White only	597 (53.9%)
Black only	116 (10.5%)
Hispanic/Latino only	34 (3.1%)
Other (including biracial)	361 (32.6%)
BMI, mean ± SD (range)	24.4 ± 4.9 (12.6–48.8)
Fruit and Vegetable Intake Score, mean ± SD (range)	2.4 ± 2.3 (0.1–14.8)
Cooking Frequency, *n* (%)	
0 times weekly/not at all	801 (73.2%)
1–3 times weekly	214 (19.6%)
4–7 times weekly	79 (7.2%)
Cooking Type	
Do not cook	474 (42.9%)
Cook mostly convenience and ready-made meals	366 (33.2%)
Cook from basic ingredients	264 (23.9%)
Cooking Confidence, mean ± SD (range)	4.8 ± 1.7 (1–7)
Self-instruction for intention of healthful mealtime behavior, mean ± SD (range)	3.3 ± 0.75 (1–5)
Self-regulation for healthful mealtime behavior, mean ± SD (range)	3.4 ± 0.84 (1–5)
Residency, *n* (%)	
On campus	962 (86.9%)
Off campus	141 (12.7%)
University, *n* (%)	
Auburn University	69 (6.2%)
University of Florida	298 (26.9%)
Maine University	164 (14.8%)
Kansas State University	111 (10.0%)
Syracuse University (New York)	145 (13.1%)
University of Tennessee	164 (14.8%)
South Dakota State University	67 (6.0%)
West Virginia University	87 (7.9%)
Working Hours, *n* (%)	
I do not work	799 (72.1%)
I do work (1 h or greater per week)	288 (26.0%)
Pell Grant Eligible ^a^, *n* (%)	
Yes	388 (35.0%)
No	659 (59.5%)
Sex, *n* (%)	
Male	368 (33.2%)
Female	736 (66.4%)

^a^ Pell Grant Eligible was used as an indicator of socioeconomic status. A Pell Grant is a USA federal subsidy for post-secondary education based on financial need of the student.

**Table 2:** Cooking and meal planning as predictors of fruit and vegetable intake and BMI in first-year college students.

Predictor ^a^	Fruit and Vegetable Intake		Body Mass Index	
	Beta Coefficient	*p*-Value	Beta Coefficient	*p*-Value
Cooking Frequency				
0 times weekly/not at all ^b^				
1–3 times weekly	0.03	0.627	−0.002	0.900
4–7 times weekly	0.26	0.004	−0.03	0.245
Cooking Type				
Do not cook ^b^				
Cook mostly convenience and ready-made meals	−0.14	0.009	−0.000	0.998
Cook from basic ingredients	0.05	0.392	−0.03	0.044
Cooking Confidence	0.03	0.055	−0.000	0.884
Self-instruction for intention of healthful mealtime behavior	0.24	0.000	0.02	0.027
Self-regulation for healthful mealtime behavior	0.34	0.000	−0.01	0.033

^a^ Multiple linear regression was used to determine associations among cooking patterns, meal planning behaviors, fruit and vegetable intake and Body Mass Index (BMI), after controlling for the influence of sex. Separate models were run for each dependent variable (log fruit and vegetable intake and log BMI) and independent variable of interest (cooking patterns and meal planning behaviors) and statistical significance was set at *p* ≤ 0.05. ^b^ The first subcategory of each variable is used as a reference category, and therefore does not have a corresponding *p*-value or Beta coefficient.
